# Supplementary material for: Causal Relationship between Aging and Anorexia Nervosa: A White-Matter-Microstructure-Mediated Mendelian Randomization Analysis
Source: Biomedicines. 2024 Aug 16;12(8):1874. doi: 10.3390/biomedicines12081874 (PMC11351342; doi:10.3390/biomedicines12081874)
Supplement: Supplementary file 1 [file biomedicines-12-01874-s001.zip › Supplementary Figure/Figure S4/Singe SNP plot of MR analysis for TL to ubm-b-2056.pdf]

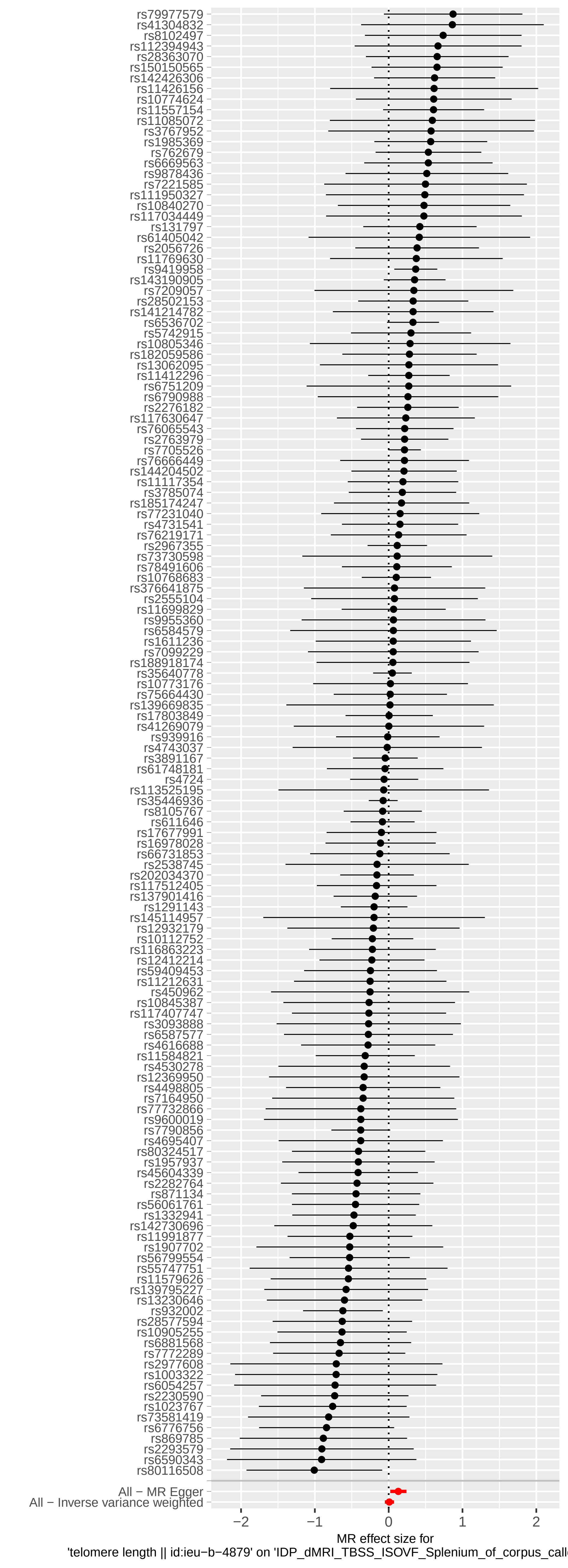
rs79977579
rs41304832
rs8102497
rs112394943
rs28363070
rs150150565
rs142426306
rs11426156
rs10774624
rs11557154
rs11085072
rs3767952
rs1985369
rs762679
rs6669563
rs9878436
rs7221585
rs111950327
rs10840270
rs117034449
rs131797
rs61405042
rs2056726
rs11769630
rs9419958
rs143190905
rs7209057
rs28502153
rs141214782
rs6536702
rs5742915
rs10805346
rs182059586
rs13062095
rs11412296
rs6751209
rs6790988
rs2276182
rs117630647
rs76065543
rs2763979
rs7705526
rs76666449
rs144204502
rs11117354
rs3785074
rs185174247
rs77231040
rs4731541
rs76219171
rs2967355
rs73730598
rs78491606
rs10768683
rs376641875
rs2555104
rs11699829
rs9955360
rs6584579
rs1611236
rs7099229
rs188918174
rs35640778
rs10773176
rs75664430
rs139669835
rs17803849
rs41269079
rs939916
rs4743037
rs3891167
rs61748181
rs4724
rs113525195
rs35446936
rs8105767
rs611646
rs17677991
rs16978028
rs66731853
rs2538745
rs202034370
rs117512405
rs137901416
rs1291143
rs145114957
rs12932179
rs10112752
rs116863223
rs12412214
rs59409453
rs11212631
rs450962
rs10845387
rs117407747
rs3093888
rs6587577
rs4616688
rs11584821
rs4530278
rs12369950
rs4498805
rs7164950
rs77732866
rs9600019
rs7790856
rs4695407
rs80324517
rs1957937
rs45604339
rs2282764
rs871134
rs56061761
rs1332941
rs142730696
rs11991877
rs1907702
rs56799554
rs55747751
rs11579626
rs139795227
rs13230646
rs932002
rs28577594
rs10905255
rs6881568
rs7772289
rs2977608
rs1003322
rs6054257
rs2230590
rs1023767
rs73581419
rs6776756
rs869785
rs2293579
rs6590343
rs80116508
All − MR Egger
All − Inverse variance weighted
−2
−1
0
1
2
MR effect size for
'telomere length || id:ieu−b−4879' on 'IDP_dMRI_TBSS_ISOVF_Splenium_of_corpus_call
